# Association of *ACTN3 R577X* but not *ACE* I/D gene variants with elite rugby union player status and playing position

**DOI:** 10.1152/physiolgenomics.00107.2015

**Published:** 2016-01-12

**Authors:** S. M. Heffernan, L. P. Kilduff, R. M. Erskine, S. H. Day, J. S. McPhee, G. E. McMahon, G. K. Stebbings, J. P. H. Neale, S. J. Lockey, W. J. Ribbans, C. J. Cook, B. Vance, S. M. Raleigh, C. Roberts, M. A. Bennett, G. Wang, M. Collins, Y. P. Pitsiladis, A. G. Williams

**Affiliations:** ^1^MMU Sports Genomics Laboratory, Manchester Metropolitan University, Crewe, United Kingdom;; ^2^A-STEM, College of Engineering, Swansea University, Swansea, United Kingdom;; ^3^Research Institute for Sport & Exercise Sciences, Liverpool John Moores University, Liverpool, United Kingdom;; ^4^Institute of Sport, Exercise and Health, University College London, London, United Kingdom;; ^5^School of Healthcare Science, Manchester Metropolitan University, Manchester, United Kingdom;; ^6^Northern Ireland Sports Institute, Newtownabbey, Belfast, United Kingdom;; ^7^Division of Sport, Exercise and Life Science, University of Northampton, Northampton, United Kingdom;; ^8^School of Sport, Health and Exercise Sciences, Bangor University, Bangor, United Kingdom;; ^9^Institute of Cardiovascular & Medical Sciences University of Glasgow, Glasgow, United Kingdom;; ^10^Medical and Scientific Department, South African Rugby Union, Cape Town, South Africa;; ^11^Discipline of Sports Science, Faculty of Health Sciences, University of Kwazulu-Natal, Durban, South Africa;; ^12^Centre for Sport and Exercise Science and Medicine (SESAME), University of Brighton, Brighton, United Kingdom; and; ^13^MRC/UCT Research Unit for Exercise Science and Sports Medicine, University of Cape Town (UCT), Cape Town, South Africa

**Keywords:** α-actinin-3, angiotensin converting enzyme, athlete genetics, RugbyGene project

## Abstract

We aimed to quantify the *ACE* I/D and *ACTN3* R577X (rs1815739) genetic variants in elite rugby athletes (rugby union and league) and compare genotype frequencies to controls and between playing positions. The rugby athlete cohort consisted of 507 Caucasian men, including 431 rugby union athletes that for some analyses were divided into backs and forwards and into specific positional groups: front five, back row, half backs, centers, and back three. Controls were 710 Caucasian men and women. Real-time PCR of genomic DNA was used to determine genotypes using TaqMan probes and groups were compared using χ^2^ and odds ratio (OR) statistics. Correction of *P* values for multiple comparisons was according to Benjamini-Hochberg. There was no difference in *ACE* I/D genotype between groups. *ACTN3* XX genotype tended to be underrepresented in rugby union backs (15.7%) compared with forwards (24.8%, *P* = 0.06). Interestingly, the 69 back three players (wings and full backs) in rugby union included only six XX genotype individuals (8.7%), with the R allele more common in the back three (68.8%) than controls (58.0%; χ^2^ = 6.672, *P* = 0.04; OR = 1.60) and forwards (47.5%; χ^2^ = 11.768, *P* = 0.01; OR = 2.00). Association of *ACTN3* R577X with playing position in elite rugby union athletes suggests inherited fatigue resistance is more prevalent in forwards, while inherited sprint ability is more prevalent in backs, especially wings and full backs. These results also demonstrate the advantage of focusing genetic studies on a large cohort within a single sport, especially when intrasport positional differences exist, instead of combining several sports with varied demands and athlete characteristics.

rugby is an intermittent team sport comprising two similar but differing codes, rugby league (RL) and rugby union (RU). Both codes consist of diverse playing positions, each with different physiological, anthropometric, and technical attributes (8, 10, 20, 27) including two distinct subgroups in each code: forwards and backs. Recently, global positioning system tracking and time-motion analysis have been used to estimate the physical demands of rugby athletes and compare forwards and backs during high-level match play (8, 20, 27). In RU, backs travelled 12% greater total distance (6,545 m vs. 5,850 m), achieved maximum speeds 16% faster (30.4 km/h vs. 26.3 km/h) and engaged in over four times (58% vs. 13%) high-intensity running activities (>5.0 m/s), as a proportion of total activity (8, 27) compared with forwards. These data suggest a more sprint-oriented metabolic demand in backs compared with forwards. Furthermore, due to the complexities of forward play, forwards performed sixfold more (9.9%) high-intensity static exertion activities (rucks, mauls, scrums, and line-outs) than backs (1.6%) and spent 19.8% more time running above 80% of their maximal speed (8, 27, respectively). This implies that forwards, although often of higher body mass (14), are more likely to benefit from fatigue-resistant physiological qualities than backs. Accordingly, Deutsch et al. (10) showed that forwards had a notably higher work-to-rest ratio than backs (1:7 and 1:22, respectively). Given that the roles of backs and forwards differ significantly in terms of physiological demands, these differences may be reflected in distinct genetic characteristics (18). Elite RL athletes cover similar total distances (∼7,000 m vs. ∼5,000 m; backs vs. forwards, respectively) and have similar anthropometric characteristics to RU athletes (20). Players regularly transfer between RL and RU codes so investigating both codes (combined and separately) for their genetic characteristics is justified.

The two most studied gene variants in exercise genomics (*ACE* I/D and *ACTN3* R577X polymorphisms) have recently been considered in meta-analyses. Ma et al. (23) reported that *ACE* II genotype was associated with physical performance [odds ratio (OR) 1.23], especially endurance performance (OR 1.35). Furthermore, *ACTN3* RR genotype was associated with speed and power performance (OR 1.21; 23), supported elsewhere (2). More extensive information regarding *ACE* I/D and *ACTN3* R577X polymorphisms is available (13, 26). Due to differences in physical characteristics between rugby athletes and the general population and the diverse physiological demands within rugby, these genetic markers could predispose athletes to success or specific roles at the elite level.

One recent paper examined *ACE* I/D genotype frequency distribution in young, nonelite RU athletes. *ACE* I/D genotype frequencies did not differ between forwards and backs, with no control group included (5). The same group (4) also investigated *ACTN3* R577X in 102 young male RU athletes and reported no association, despite some tendencies for the R allele to be more frequent in backs or subgroups of backs. Studying elite athletes would be better able to answer the question whether these genetic variants are associated with elite status and playing position in rugby.

Therefore, the purpose of the present study was to investigate whether elite rugby athletes in the RugbyGene project (18) and a control group differed in terms of *ACE* I/D and *ACTN3* R577X genotype distribution and whether athletes in specialized playing positions similarly differed. It was hypothesized that the *ACTN3* R allele and the *ACE* I allele would be more frequent in rugby athletes than controls. It was further hypothesized that *ACTN3* XX and *ACE* II genotypes would be underrepresented in RU backs compared with forwards, due to differences in overall work-to-rest ratio and differing requirements for high maximum speed.

## METHODS

### Participants

Ethical approval was granted by Manchester Metropolitan University (MMU), University of Glasgow, University of Cape Town, and Northampton University ethics committees, and the study complies with the Declaration of Helsinki. As part of the RugbyGene project, elite Caucasian male rugby athletes [*n* = 507; mean (standard deviation) height 1.85 (0.07) m, mass 101 (14) kg, age 29 (7) yr] including 71.2% British, 17.2% South African, 7.1% Irish, and 4.5% of other nationalities were recruited, having given written informed consent. Caucasian controls [61% male; *n* = 710; height 1.73 (0.10) m, mass 74 (13) kg, age 29 (16) yr] included 89.6% British, 8.9% South African, 0.7% Irish, and 0.8% of other nationalities. Athletes were considered elite if they had competed regularly (>5 matches) since 1995 in the highest professional league in the UK, Ireland, or South Africa for RU and the highest professional league in the UK for RL. Of the RU athletes, 53.4% had competed at international level for a “High Performance Union” (Regulation 16, http://www.worldrugby.org), and 38.5% of RL had competed at international level. International status was confirmed as of 1 January 2015. Athletes were taller and heavier (*P* < 0.0005) but not older (*P* = 0.871) than controls.

### Procedures

#### Sample collection.

Blood (∼70% of all samples), saliva (∼25%), or buccal swab samples (∼5%) were obtained via the following protocols. Blood was drawn from a superficial forearm vein into an EDTA tube and stored in sterile tubes at −20°C until processing. Saliva samples were collected into Oragene DNA OG-500 collection tubes (DNA Genotek, Ottawa, Ontario, Canada) according to the manufacturer's protocol and stored at room temperature until processing. Sterile buccal swabs (Omni swab; Whatman, Springfield Mill, UK) were rubbed against the buccal mucosa of the cheek for ∼30 s. Tips were ejected into sterile tubes and stored at −20°C until processing.

#### DNA isolation and genotyping.

DNA isolation and genotyping were performed in the MMU, University of Glasgow, University of Cape Town (DNA isolation only), and University of Northampton laboratories. There are some differences between protocols summarized below; however, there was 100% agreement among reference samples genotyped in the three genotyping centers, i.e., Glasgow, Northampton, and MMU laboratories. The majority of samples were processed and genotyped in the MMU laboratory. Genotype calling was successful for both variants in all samples.

At MMU and Glasgow, DNA isolation was performed with the QIAamp DNA Blood Mini kit and standard spin column protocol, following the manufacturer's instructions (Qiagen, West Sussex, UK). Briefly, 200 μl of whole blood/saliva, or one buccal swab, was lysed and incubated, the DNA washed, and the eluate containing isolated DNA stored at 4°C. In Cape Town, DNA was isolated from whole blood by a different protocol (22). In brief, samples were lysed and centrifuged, the DNA washed, and samples stored at −20°C. Genotyping of DNA isolated in Cape Town was performed in Glasgow. At Northampton, DNA was isolated from whole blood with Flexigene kits (Qiagen). In brief, samples were lysed, and DNA precipitated and washed, with samples stored at −20°C.

#### Genotyping.

Genotyping in the Glasgow laboratory was performed on *ACTN3* (rs1815739) and an *ACE* tag single nucleotide polymorphism (SNP) (rs4341) in perfect linkage disequilibrium with *ACE* I/D in Caucasians (15). In brief, 10 μl Genotyping Master Mix (Applied Biosystems, Paisley, UK), 1 μl SNP-specific TaqMan assay (Applied Biosystems), 6 μl nuclease-free H_2_O, and 3 μl DNA solution (∼9 ng DNA) were added per well. In the Northampton laboratory, genotyping was performed for *ACTN3* R577X (rs1815739) by combining 10 μl of Genotyping Master Mix, 8 μl H_2_O, 1 μl assay mix with 1 μl of purified DNA (∼10 ng). In both laboratories, PCR was performed using a StepOnePlus real-time detector (Applied Biosystems). In brief, denaturation began at 95°C for 10 min, with 40 cycles of incubation at 92°C for 15 s then annealing and extension at 60°C for 1 min. Initial analysis was performed using StepOnePlus software version 2.3 (Applied Biosystems). There was 100% agreement within duplicates of all samples.

At MMU, samples were genotyped for *ACTN3* R577X (rs1815739) by combining 5 μl Genotyping Master Mix, 4.3 μl H_2_O, 0.5 μl assay mix, and 0.2 μl of purified DNA (∼9 ng), for samples derived from blood and saliva. For DNA derived from buccal swabs, 5 μl Genotyping Master Mix was combined with 3.5 μl H_2_O, 0.5 μl assay mix, and 1 μl DNA solution (∼9 ng DNA). Either a Chromo4 real-time system (Bio-Rad, Hertfordshire, UK) or a StepOnePlus was used. In brief, denaturation began at 95°C for 10 min, with 40 cycles of incubation at 92°C for 15 s and then annealing and extension at 60°C for 1 min. Initial genotyping analysis was performed with Opticon Monitor software version 3.1 (Bio-Rad) or StepOnePlus software version 2.3. Duplicates of all samples were in 100% agreement. For *ACE* I/D at MMU, 5 μl of Genotyping Master Mix, 1.55 μl H_2_O, 0.9 μl of I and D allele-specific probes, and 0.38 μl of *ACE* primer 111, 112, and 113 (sequences below) were combined with 0.5 μl DNA solution (∼23 ng DNA) per well for blood and saliva. For DNA derived from buccal cells, primer and probe volumes were identical, but 0.05 μl H_2_O and 2 μl DNA solution (∼18 ng DNA) were used. Similarly, in the Northampton laboratory, *ACE* I/D was genotyped by combining 11 μl of Genotyping Master Mix, 2 μl of I and D probes, 2 μl of *ACE* primer 111, 112, 113, and 4 μl DNA solution (∼40 ng DNA). Either a Chromo4 real-time system or a StepOnePlus was used. In brief, there were 50 cycles of denaturation at 92°C for 15 s and then annealing and extension at 57°C for 1 min. Initial analysis was performed with Opticon Monitor 3.1 software or StepOnePlus software version 2.3. Again, there was 100% agreement within duplicates of all samples.

#### Primers and probes.

For rs1815739 and rs4341, the appropriate TaqMan assay was used (Applied Biosystems). For the direct *ACE* I/D assay, three primers (150 nM each) and probes (VIC, 150 nM and FAM, 75 nM; 21) were used: primer ACE111, 5′-CCCATCCTTTCTCCCATTTCTC-3′; primer ACE112, 5′-AGCTGGAATAAAATTGGCGAAAC-3′; primer ACE113, 5′-CCTCCCAAAGTGCTGGGATTA-3′; I allele-specific probe (VIC-ACE100), VIC-5′-AGGCGTGATACAGTCA-3′-MGB; D allele-specific probe (FAM-ACE100), FAM-5′-TGCTGCCTATACAGTCA-3′-MGB.

### Positional Groups

To assess genotype and allele frequencies within the RU group, we allocated athletes to subgroups: forwards (props, hookers, locks, flankers, number eights) and backs (scrum halves, fly halves, centers, wings, full backs). Also, due to diverse physiological demands within RU (8, 27), athletes were further divided into positional groups according to their similar movement patterns (8) front five (props, hookers, locks), back row (flankers, number eights), half backs (scrum halves, fly halves), centers, and back three (wings and full backs). Comparisons between positions were not performed for the RL cohort due to low statistical power when it was subdivided.

### Data Analysis

SPSS for Windows version 19 (SPSS, Chicago, IL) software was used to conduct Pearson's Chi-square (χ^2^) tests to compare genotype and allelic frequencies between athletes and controls and between positional subgroups. For *ACTN3* and *ACE*, 26 and 16 tests, respectively, were subjected to Benjamini-Hochberg (BH; 6) corrections to control false discovery rate, and corrected probability values are reported. Where appropriate, OR was calculated to estimate effect size. Alpha was set at 0.05.

## RESULTS

**Fig. 1.**
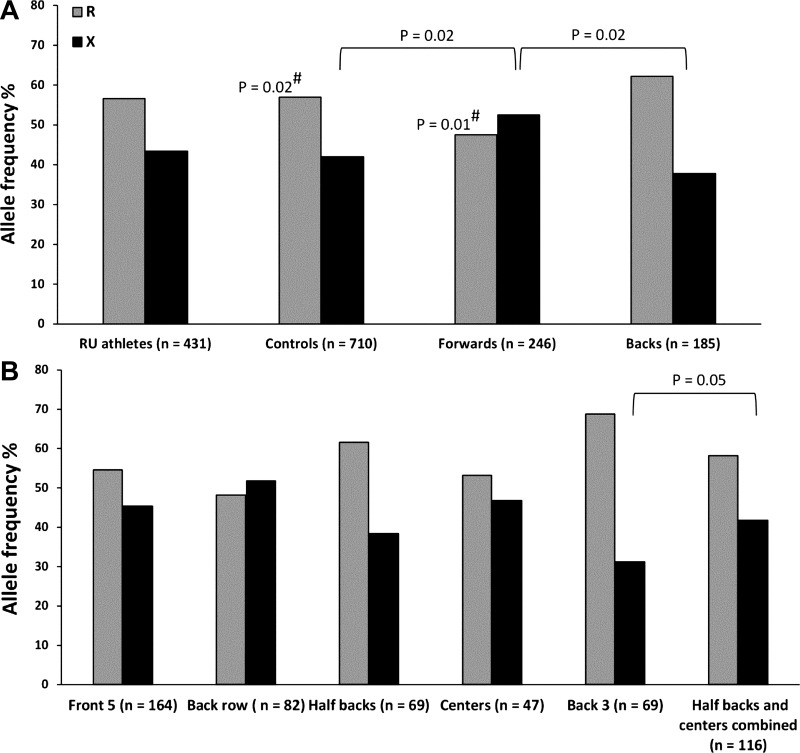
*ACTN3* allele frequencies. *A*: allele frequencies of rugby union (RU) athletes and controls, with athletes also divided into playing subgroup (forwards and backs). #Different from the back 3. *B*: allele frequencies of RU athletes divided into positional groups with the addition of the “half backs and centers combined” group. Statistical analysis between these positional groups only compared the back 3 with the half backs and centers combined.

All genotype data for athletes and controls were in Hardy-Weinberg equilibrium. There were no differences in genotype frequencies within the athlete or control groups according to nationality. For *ACE* I/D, there were no differences between all athletes (RU and RL combined) and controls in genotype (χ^2^ = 1.117, *P* = 0.83), between RU or RL and controls, nor between playing subgroups for RU (Table 1). Furthermore, for *ACTN3* R577X there were no genotype differences between controls and all athletes (χ^2^ = 1.645, *P* = 0.44), RL (χ^2^ = 1.829, *P* = 0.44), or RU (χ^2^ = 0.216, *P* = 0.33). However, when we considered RU playing position, the X allele was overrepresented in forwards (52.5%) compared with backs [37.8%, χ^2^ = 8.128, *P* = 0.02; OR = 1.49, 95% confidence interval (CI) = 1.13–1.96, *P* = 0.004] and controls (42%, χ^2^ = 6.217, *P* = 0.02; OR = 1.25, 95% CI = 1.02–1.54, *P* = 0.033; Table 1 and Fig. 1*A*). Similarly, there was a tendency (*P* = 0.023 before BH correction) of the XX genotype to be overrepresented in forwards (24.8%) compared with backs (15.7%, χ^2^ = 5.193, *P* = 0.08; OR = 1.77, 95% CI = 1.09–2.89, *P* = 0.022) and controls (18.3%, χ^2^ = 7.582, *P* = 0.08), with no difference between backs and controls (χ^2^ = 3.043, *P* = 0.37).

**Table 1.** Genotype and allele distribution of controls and athletes divided into positional subgroups (for RU only), presented as genotype/allele counts followed by percentage in parentheses

Genotype	All Athletes	RL Athletes	RU Athletes	Controls	Forwards	Front 5	Back Row	Backs	Half Backs	Centers	Back 3
*ACE*											
II	108 (21.4)	18 (21.7)	92 (21.5)	113 (19.8)	49 (20.0)	36 (22.1)	13 (15.9)	43 (23.6)	14 (20.3)	14 (31.1)	15 (22.1)
ID	251 (49.7)	39 (47.0)	214 (50.1)	286 (50.0)	129 (52.7)	86 (52.8)	43 (52.4)	85 (46.7)	33 (47.8)	17 (37.8)	35 (51.5)
DD	146 (28.9)	26 (31.3)	121 (28.3)	172 (30.2)	67 (27.3)	41 (25.2)	26 (31.7)	54 (29.7)	22 (31.9)	14 (31.1)	18 (26.5)
Total	505	83	427	572	245	163	82	182	69	45	68
I allele	467 (46.3)	75 (45.2)	398 (46.6)	512 (44.7)	227 (46.3)	158 (48.5)	69 (42.1)	171 (47.0)	61 (44.2)	45 (50.0)	65 (47.8)
D allele	543 (53.7)	91 (54.8)	456 (53.4)	630 (55.3)	263 (53.7)	168 (51.5)	95 (57.9)	193 (53.0)	77 (55.8)	45 (50.0)	71 (52.2)
*ACTN3*											
XX	104 (20.5)	15 (18.1)	90 (20.9)	130 (18.3)	61 (24.8)	39 (23.8)	22 (26.8)	29 (15.7)	12 (17.4)	11 (23.4)	6 (8.7)*
RX	234 (46.2)	45 (54.2)	194 (45.0)	337 (47.5)	112 (45.5)	71 (43.3)	41 (50.0)	82 (44.3)	29 (42.0)	22 (46.8)	31 (44.9)
RR	169 (33.3)	23 (27.7)	147 (34.1)	243 (34.2)#	73 (29.7)#	54 (32.9)	19 (23.2)	74 (40.0)	28 (40.6)	14 (29.8)	32 (46.4)
Total	507	83	431	710	246	164	82	185	69	47	69
X allele	442 (43.5)	75 (45.2)	374 (43.4)	597 (42.0)*	234 (47.6)	149 (45.4)	85 (51.8)	140 (37.8)*	53 (38.4)	44 (46.8)	43 (31.2)
R allele	572 (56.5)	91 (54.8)	488 (56.6)	823 (58.0)#	258 (52.4)	179 (54.6)	79 (48.2)	230 (62.2)	85 (61.6)	50 (53.2)	95 (68.8)*

RL, rugby league; RU, rugby union.

* Different from forwards.

# Different from the Back 3.

Interestingly, the 69 back three athletes (wings and fullbacks) included only six individuals (8.7%) of XX genotype that differed from the forwards (24.8%; χ^2^ = 11.082, *P* = 0.05; OR = 3.46, 95% CI = 1.43–8.34, *P* = 0.006) and tended to differ from the combined half backs and centers group (19.8%; χ^2^ = 4.151, *P* = 0.08; OR = 2.59, 95% CI = 1.00–6.74, *P* = 0.049). Likewise, the R allele distribution was greater in the back three (68.8%) than the controls (58.0%; χ^2^ = 6.672, *P* = 0.02; OR = 1.60, 95% CI = 1.09–2.33, *P* = 0.014), forwards (47.5%; χ^2^ = 11.768, *P* = 0.01; OR = 2.00, 95% CI = 1.34–2.99, *P* = 0.0007), and the other backs (58.2%; χ^2^ = 4.173, *P* = 0.05; OR = 1.59, 95% CI = 1.02–2.48, *P* = 0.042) (Fig. 1*B*).

## DISCUSSION

The present study is the first to show a genetic association with elite athlete status in RU. We found associations for the *ACTN3* R577X polymorphism but not for *ACE* I/D, thus rejecting our hypotheses regarding *ACE* I/D. Furthermore, no difference was observed for the *ACTN3* R577X genotype or allele distribution between all athletes and controls, thus rejecting the hypothesis that differences would exist between nonathletes and all players as a single cohort. Similarly, there were no differences between the RU, RL, and control groups when playing position was not considered. However, as hypothesized, in RU backs compared with forwards there was a lower proportion of XX genotype and X allele, which probably reflects the greater need for speed generation in backs and more sustained activity in forwards. The small cohort of RL athletes means that comparisons between playing positions are not feasible until the cohort increases substantially.

### ACTN3 R577X

The most remarkable finding of the present study was the low frequency of the XX genotype among the back three RU athletes (8.7%), approaching, although not as low as, the frequency observed in elite sprinters (25, 31). The XX genotype is present in ∼18% of Caucasians (Table 1) and indicates absence of the α-actinin-3 protein (3, 24). Absence of α-actinin-3, a protein almost exclusively expressed in fast-twitch skeletal muscle fibers, could hinder back three (wing and full back) sprint ability. R allele carriers have a greater proportion of type II and IIx fibers and larger relative surface area per IIx fiber than XX carriers (1, 7, 30). Furthermore, Seto et al. (29) recently showed the likely mechanism for this genotype-phenotype association is via the calcineurin muscle fiber remodeling pathway. They found greater calcineurin activity (which induces slow myogenic programming and a shift toward oxidative phenotype) in α-actinin-3 knockout mice (KO) and humans (*ACTN3* 577XX genotype) due to preferential binding of α-actinin-2 (upregulated in the absence of α-actinin-3) to the fast fiber-specific calsarcin-2 (an inhibitor of calcineurin). This could explain the advantage of R allele carriers over α-actinin-3-deficient XX individuals for high-velocity contractions, particularly important for back three RU players. While backs and forwards previously showed similar fiber type proportions (19), these older data are arguably not relevant to modern rugby athletes, given their changed physical characteristics in recent years (14). Skeletal muscle fiber type proportions are unknown in contemporary elite RU athletes who now compete in a more popular, fully professional sport and complete much higher training loads than previously. Recent in vivo data also show that R allele carriers exhibit greater muscle volume and maximal power output (11, 17). While RU forwards show greater maximal power, backs are able to generate greater power relative to body mass (W/kg; 9), which corresponds with the greater R allele frequency in the backs and especially the back three players. These data, plus evidence that type II fibers are larger and more powerful per unit volume than type I (15), suggest the R allele would benefit back three rugby athletes for muscle power and fast fiber characteristics, which supports our findings (Table 1 and Fig. 1).

Arguably, the higher propensity for aerobic enzyme activity (porin, COX IV, hexokinase, citrate synthase, succinate dehydrogenase, and β-hydroxyacyl CoA dehydrogenase; 28, 29) and greater force recovery after fatigue observed in α-actinin-3-deficient mice (28) could indicate that XX genotype humans might have a greater capacity for recovery from fatiguing exercise, a trait that would benefit forwards with their more sustained match play intensity and necessity for quick recovery. The shorter rest periods for forwards compared with backs (work-to-rest ratios 1:7.4 and 1:21.8, respectively; 10) indicates that greater fatigue resistance would be particularly beneficial for forwards. Moreover, the greater calcineurin activity in XX homozygote humans and approximately threefold increase in calcineurin activity and distance run after endurance training in KO mice (29) further support the notion that forwards would benefit from a greater fatigue resistance, especially with exposure to extensive training. These data are consistent with our observation that forwards exhibit higher XX genotype and lower R allele frequencies than backs and controls (Table 1).

When many sports were considered simultaneously, team sport athlete status showed no association with *ACTN3* R577X genotype (12). However, when one considers the relatively small number of athletes (205) with mixed status (56.6% elite) from a range of sports (ice hockey, handball, soccer, etc.), that is perhaps not surprising. While combining cohorts from different sports can boost sample size and theoretically increase statistical power, if an association does not exist in all sports, or even in all athletes within a particular sport due to positional differences, one would be less likely to detect an association. The positional differences identified within the present study demonstrate the value of studying a large sample from a single sport and, in the absence of detailed physiological data (often difficult to obtain from large numbers of elite athletes), provide a viable alternative for future genetic research involving team sport athletes.

### ACE I/D

The current study reports no difference between rugby athletes and controls or any positional subgroups for *ACE* I/D. This lack of association contrasts with a recent meta-analysis where the *ACE* I allele was associated with physical performance (23). However, Ma et al. (23) also report that specialized distance/endurance athletes showed the strongest association with the I allele (OR 1.35). Given the mixed metabolic nature of rugby, a comparable association in the present study was less likely. Furthermore, the importance of *ACE* I/D remains controversial in the literature, with no associations reported in other isolated team sports such as elite European soccer (16) and nonelite RU (5). These prior data, in conjunction with our current findings in a larger study that also considers playing position, suggest that *ACE* I/D plays little role in performance of team sport athletes. *ACE* I/D genotype-athlete phenotype associations are more likely to exist in specialized endurance athletes (26).

### Effect Size and Future Applications

OR were calculated to estimate the likelihood that individuals with the advantageous genotype/allele become an elite RU athlete in a specific position. The *ACTN3* XX genotype was almost twice (OR = 1.77) as common in forwards than backs, which suggests α-actinin-3-deficient individuals are more suited to forward play. Furthermore, forwards were over three times (OR = 3.46) more likely to be XX genotype than the back three athletes, while the remaining backs (centers and halves) were over twice as likely to show the α-actinin-3-deficient genotype than the back three (OR = 2.59). These data suggest the *ACTN3* R577X polymorphism shows potential to contribute to position-specific player profiling within RU when combined with other genetic and physiological data in the future. In contrast, the *ACE* I/D polymorphism (OR ∼1) does not show equivalent potential in rugby.

While the present cohort size is large compared with previous single sport genetic analyses, when the cohort was subdivided into playing position, the numbers were reduced so enlargement of our cohort and replication would be welcome. Accordingly we continue to recruit elite RU and RL players in the RugbyGene project and so will steadily become better able to investigate genetic aspects of specific demands within rugby. To conclude, the present study revealed position-specific genetic variation in elite RU athletes for *ACTN3* R577X. The R allele was an advantage for backs, particularly the back three. Moreover, the current results do not support *ACE* I/D as a genetic marker for rugby performance, showing no differences between athletes and controls or positional subgroups. This study demonstrates the value of single sport cohorts and the need for large sample sizes when conducting gene association studies in sport. Future objectives of the RugbyGene project within the broader Athlome project include investigating whether genetic variants associated with excellence in other sports are similarly associated in the multifaceted sport of rugby.

## DISCLOSURES

No conflicts of interest, financial or otherwise, are declared by the author(s).

## AUTHOR CONTRIBUTIONS

Author contributions: S.M.H., L.P.K., R.M.E., S.H.D., M.C., Y.P.P., and A.G.W. conception and design of research; S.M.H., R.M.E., J.S.M., G.K.S., J.P.H.N., S.J.L., M.C., and A.G.W. performed experiments; S.M.H., R.M.E., J.S.M., G.K.S., J.P.H.N., S.J.L., M.C., and A.G.W. analyzed data; S.M.H., L.P.K., R.M.E., S.H.D., J.S.M., G.E.M., G.K.S., J.P.H.N., S.J.L., W.J.R., C.J.C., B.V., S.M.R., C.R., M.A.B., G.W., M.C., Y.P.P., and A.G.W. interpreted results of experiments; S.M.H. prepared figures; S.M.H. and C.R. drafted manuscript; S.M.H., L.P.K., R.M.E., S.H.D., J.S.M., G.E.M., G.K.S., J.P.H.N., S.J.L., W.J.R., C.J.C., B.V., S.M.R., C.R., M.A.B., G.W., M.C., Y.P.P., and A.G.W. edited and revised manuscript; S.M.H., L.P.K., R.M.E., S.H.D., J.S.M., G.E.M., G.K.S., J.P.H.N., S.J.L., W.J.R., C.J.C., B.V., S.M.R., C.R., M.A.B., G.W., M.C., Y.P.P., and A.G.W. approved final version of manuscript.
